# Heme-Oxygenase-1 Expression Contributes to the Immunoregulation Induced by *Fasciola hepatica* and Promotes Infection

**DOI:** 10.3389/fimmu.2017.00883

**Published:** 2017-07-26

**Authors:** Paula Carasi, Ernesto Rodríguez, Valeria da Costa, Sofía Frigerio, Natalie Brossard, Verónica Noya, Carlos Robello, Ignacio Anegón, Teresa Freire

**Affiliations:** ^1^Laboratorio de Inmunomodulación y Desarrollo de Vacunas, Facultad de Medicina, Departamento de Inmunobiología, Universidad de República, Montevideo, Uruguay; ^2^Departamento de Bioquimica, Facultad de Medicina, Universidad de la República, Montevideo, Uruguay; ^3^Unidad de Biología Molecular, Institut Pasteur de Montevideo, Montevideo, Uruguay; ^4^Centre de Recherche en Transplantation et Immunologie UMR1064, INSERM, Université de Nantes, CHU Nantes, Nantes, France; ^5^Institut de Transplantation Urologie Néphrologie (ITUN), CHU Nantes, Nantes, France

**Keywords:** helminth, heme-oxigenase-1, immune regulation, dendritic cell, macrophage

## Abstract

*Fasciola hepatica*, also known as the liver fluke, is a trematode that infects livestock and humans causing fasciolosis, a zoonotic disease of increasing importance due to its worldwide distribution and high economic losses. This parasite immunoregulates the host immune system by inducing a strong Th2 and regulatory T immune response by immunomodulating dendritic cell (DC) maturation and alternative activation of macrophages. In this paper, we show that *F. hepatica* infection in mice induces the upregulation of heme-oxygenase-1 (HO-1), the rate-limiting enzyme in the catabolism of free heme that regulates the host inflammatory response. We show and characterize two different populations of antigen presenting cells that express HO-1 during infection in the peritoneum of infected animals. Cells that expressed high levels of HO-1 expressed intermediate levels of F4/80 but high expression of CD11c, CD38, TGFβ, and IL-10 suggesting that they correspond to regulatory DCs. On the other hand, cells expressing intermediate levels of HO-1 expressed high levels of F4/80, CD68, Ly6C, and FIZZ-1, indicating that they might correspond to alternatively activated macrophages. Furthermore, the pharmacological induction of HO-1 with the synthetic metalloporphyrin CoPP promoted *F. hepatica* infection increasing the clinical signs associated with the disease. In contrast, treatment with the HO-1 inhibitor SnPP protected mice from parasite infection, indicating that HO-1 plays an essential role during *F. hepatica* infection. Finally, HO-1 expression during *F. hepatica* infection was associated with TGFβ and IL-10 levels in liver and peritoneum, suggesting that HO-1 controls the expression of these immunoregulatory cytokines during infection favoring parasite survival in the host. These results contribute to the elucidation of the immunoregulatory mechanisms induced by *F. hepatica* in the host and provide alternative checkpoints to control fasciolosis.

## Introduction

Fasciolosis, a helminth infection caused by *Fasciola hepatica*, is of paramount importance due to its wide spectrum of definitive hosts (1) and its worldwide distribution (2) affecting both livestock and human health. World Health Organization (WHO) estimates that at least 2.4 million people are infected in more than 70 countries worldwide, with several million at risk. Several studies have independently demonstrated that *F. hepatica*-derived molecules inhibit or decrease dendritic cell (DC) activation, which results in the induction of a tolerogenic phenotype (3–7). Furthermore, we have demonstrated that DCs from mice infected with *F. hepatica* have a semi-mature phenotype that is characterized by low MHC II and CD40 expression, high secretion of the immunoregulatory cytokine IL-10, and the ability to differentiate and expand IL-10-producing CD4 T cells (8). In addition, different groups have reported that *F. hepatica*-derived molecules also modulate macrophage activation, inducing the alternative activation of IL-10-producing macrophages (9, 10) and inhibiting the production of pro-inflammatory cytokines, such as IL-1β (11), IL-10 (12), Arg-1, PDL-1 (13), and PDL-2 (14, 15). Thus, it has been hypothesized that *F. hepatica* may modulate both macrophages and DC function and fate as a mean to control its pathogenesis and survival in the infected hosts.

Heme-oxygenase-1 (HO-1), the rate-limiting enzyme in the catabolism of free heme, is involved in many physiological and pathophysiological processes, by affording cytoprotection (16) and regulating the host inflammatory response. Indeed, HO-1 is a stress-responsive enzyme important for defense against oxidant-induced injury during inflammatory processes and is highly inducible by a variety of stimuli, such as LPS, cytokines, heat shock, heavy metals, oxidants, and its substrate heme. Several works confirm that HO-1 plays a role in different infectious diseases, and can have both beneficial and detrimental consequences for the host immunity against pathogens (17). For instance, HO-1 is able to promote *Plasmodium* liver infection (18), whereas it plays a favorable role in the host during cerebral malaria (19). On the other hand, HO-1 controls a variety of infections in mice, including *Mycobacterium avium* (20), *Listeria monocytogenes* (21), *Plasmodium falciparum* (22), *Salmonella typhimurium* (23), *Toxoplasma gondii* (24), and respiratory syncytial virus (25).

Expression of HO-1 in monocyte-derived DC inhibits LPS-induced maturation and reactive oxygen species production (26). In addition, HO-1^+^ DCs express the anti-inflammatory cytokine IL-10 resulting in the inhibition of alloreactive T-cell proliferation (26). Also, IL-10-producing anti-inflammatory macrophages (M2) express HO-1 (27). Thus, HO-1 has been proposed to be key mediator of the anti-inflammatory effects of macrophages and DCs.

In the present study, we demonstrate that during infection with the trematode *F. hepatica*, HO-1 is upregulated by immune cells expressing F4/80 in the peritoneal cavity and liver. We also show that the pharmacological induction of HO-1 with the synthetic metalloporphyrin CoPP promotes *F. hepatica* infection increasing the clinical signs associated with the disease, such as liver damage. Moreover, treatment with the HO-1 inhibitor SnPP protected from parasite infection. The increase of HO-1 during *F. hepatica* infection was associated with the increase of TGFβ and IL-10 in liver and peritoneal exudate cells (PECs). Interestingly, we identified two different F4/80^+^ cell populations that expressed HO-1. HO-1^hi^ F4/80^int^ cells were characterized by the expression of CD11c, CD38, TGFβ, and IL-10 suggesting that they correspond to regulatory DCs. On the other hand, HO-1^int^ F4/80^hi^ cells expressed high levels of CD68, Ly6C, and FIZZ-1 indicating that they might be alternatively activated macrophages. Our results contribute to the elucidation of immunoregulatory mechanisms induced by *F. hepatica* in the host and could provide alternative checkpoints to control fasciolosis.

## Materials and Methods

### Ethics Statement

Mouse experiments were carried out in accordance with strict guidelines from the National Committee on Animal Research (Comisión Nacional de Experimentación Animal, CNEA, National Law 18.611, Uruguay) according to the international statements on animal use in biomedical research from the Pan American Health Organization and WHO. The protocol was approved by the Uruguayan Committee on Animal Research. Cattle’s livers were collected during the routine work of a local abattoir (Frigorífico Carrasco) in Montevideo (Uruguay).

### Mice

Six- to eight-week-old female BALB/c mice were obtained from DILAVE Laboratories (Uruguay). Animals were kept in the animal house (URBE, Facultad de Medicina, UdelaR, Uruguay) with water and food supplied *ad libitum*. Mouse handling and experiments were carried out in accordance with strict guidelines from the National Committee on Animal Research (CNEA, Uruguay). All procedures involving animals were approved by the Universidad de la República’s Committee on Animal Research (CHEA Protocol Number: 070153-000180-16).

### Infections and Cell Cultures

BALB/c mice were orally infected with 10 *F. hepatica* metacercariae (Baldwin Aquatics, USA) per animal. After 1, 2, or 3 weeks post-infection (wpi) mice were bled and PECs, spleens, and livers were removed. In order to evaluate the severity of the infection, a disease severity score was developed (Table 1), which was applied in blinded experiments by two independent experimenters. Alanine aminotransferase (ALT) activity in sera was determined by using a commercial kit (Spinreact, Spain) according to the manufacturers’ instructions. PECs from infected and non-infected mice were washed twice with PBS containing 2% FBS and 0.1% sodium azide. The following antibodies were used in these experiments anti-CD11c (N418), -I-A/I-E (2G9), CD40 (HM40-3), -F4/80 (BM8), -CD11b (M1/70), -CD172a (P84), -Ly6C (HK1.4), and -Siglec-F (E50-2440). Cells were then washed twice with PBS containing 2% FBS and 0.1% sodium azide and fixed with 1% formaldehyde. Cell populations were analyzed using a BD FACSCalibur (BD-Biosciences) or Cyan (Beckman Coulter). Expression of HO-1 (ab13248) and CD68 (FA-11) were analyzed by intracellular staining. Antibodies were obtained from Affymetrix (CA, USA), from BD-Biosciences (CA, USA), from Biolegend (CA, USA) or from Abcam.

**Table 1:** Clinical score of *Fasciola hepatica*-infected mice.

Ascites		Spleen		Number of lesions/hepatic lobe		Liver lobes	
Score	Description	Score	Size	Score	Description	Score	Description
0	None (normal cell content)	0	Normal	0	None	0	Healthy
1	Mild (medium cell content)	1	Splenomegaly (<2×)	1	<3 lesions	1	1hepatic lobe affected
2	Moderate (high cell content)	2	Splenomegaly (>2×)	2	>3 lesions	2	>2 hepatic lobes affected
3	Severe (high cell and blood content)			3	Complete affection of lobes		

*Maximal score is 10*.

### Pharmacological Induction or Inhibition of HO-1

In order to modulate HO-1 activity, mice infected with five metacercariae also received intraperitoneal injections of either vehicle (PBS, 100 µL), CoPP (20 mg/kg), SnPP (40 mg/kg), or CoPP plus SnPP. The doses of CoPP and SnPP were within a range of doses used in studies describing upregulation of HO-1 by CoPP and inhibition of the enzyme’s activity by SnPP (28, 29). Mice were injected 1 day before infection, 1 day after infection and every 5 days until the end of the experimental protocol.

### Quantitative Real-time RT-PCR

Total RNA was isolated by use of TRI-reagent (Sigma-Aldrich) from spleen, liver, PEC and purified F4/80^int^ and F4/80^hi^ cells from PEC. Samples were analyzed in an Eco real-time PCR System (Illumina) using Fast SYBR^®^ Green Master Mix (Applied Biosystems). The reactions were performed according to the following settings: 95°C for 5 min for initial activation, followed by 40 thermal cycles of 10 s at 95°C and 30 s at 60°C. All reactions were performed with at least five biological replicates.

### Microscopy Analyses

Livers from infected mice after 3 wpi or non-infected mice (control) were harvested, embedded in OCT, and snap frozen in nitrogen. Sections were cut at a thickness of 6 µm, fixed with cold acetone for 10 min and blocked with 5% BSA in 3% rat serum for 1 h at room temperature. Sections were then overnight incubated at 4°C with anti-HO-1 (ab13248) and -F4/80 (BM8), stained with DAPI and visualized in an epifluorencense microscope Olympus IX-81 and confocal microscope Leica TCS-SP5-II. The same procedure and the same antibody were used to evaluate HO-1 expression in bovine livers from naturally infected and non-infected cattle. In this case, livers were first examined by the veterinary inspector at the abattoir and determined to be infected by the presence of multiple parasites found in the bile ducts. Livers from non-infected animals were identified by absence of liver damage and flukes.

### Statistical Analysis

Results were analyzed using GraphPad Prism software (GraphPad Software, San Diego, CA, USA). Normality distribution was evaluated using the D’Agostino-Pearson omnibus normality test followed by one-way ANOVA with Bonferroni Multiple Comparison test or a student’s T test was used. Results were considered to be significantly different when *p* < 0.05 (*), 0.01 (**), or 0.001 (***).

## Results

### HO-1 Expression Is Induced in *F. hepatica*-Infected Animals

**Figure 1:**
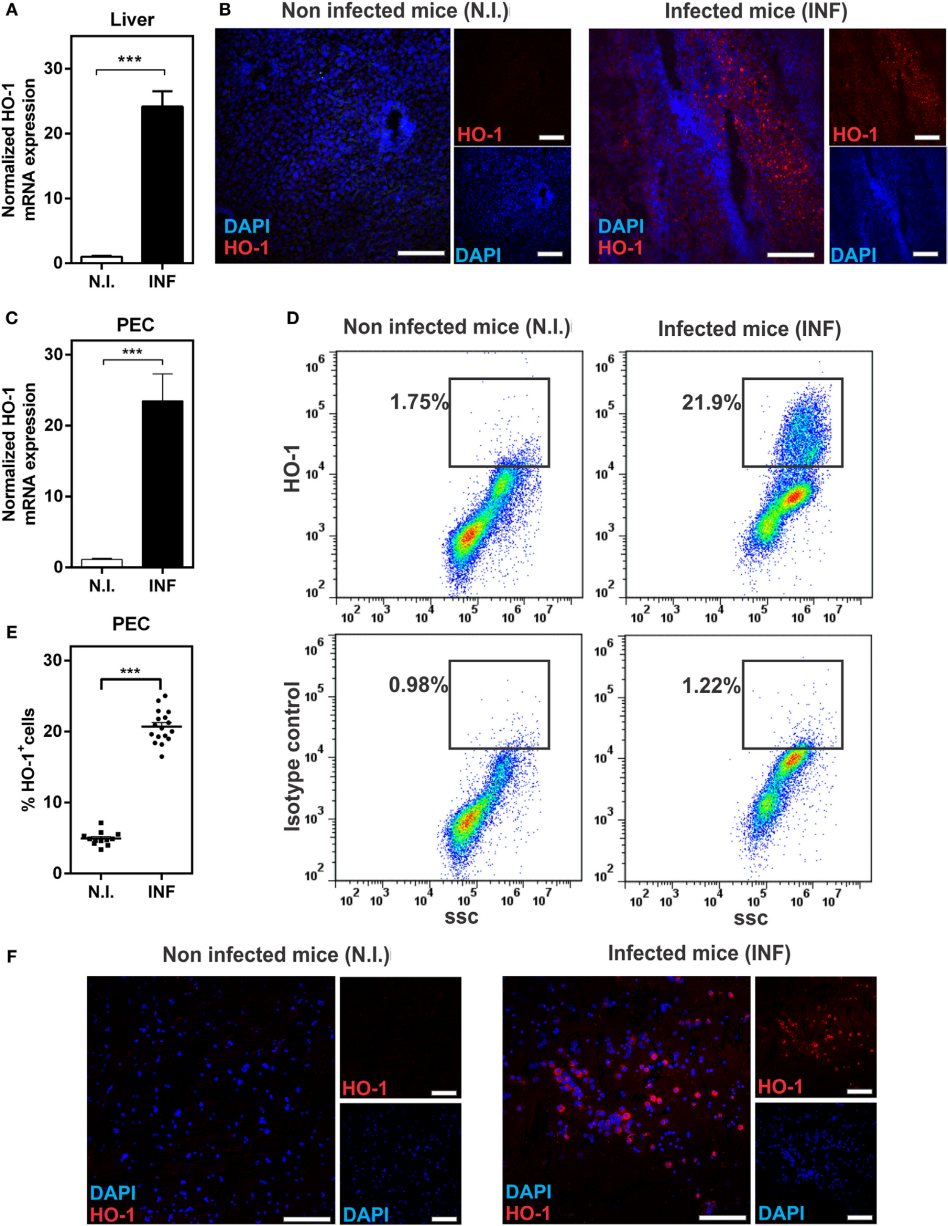
Heme-oxygenase-1 (HO-1) is induced during *Fasciola hepatica* experimental infection in mice. **(A)** mRNA expression of HO-1 in the liver from control and *F. hepatica*-infected mice at 3 wpi. **(B)** HO-1 expression in the liver from control and infected mice at 3 wpi by confocal microscopy. **(C)** mRNA expression of HO-1 in peritoneal exudate cell (PECs) from control and infected mice at 3 wpi. **(D)** HO-1^+^ cells in PECs from control and infected mice at 3 wpi by flow cytometry. **(E)** Percentage of HO-1^+^ cells in PECs from control and infected mice at 3 wpi by flow cytometry. **(F)** HO-1 expression in PECs from control and infected mice at 3 wpi by confocal microscopy. **(F)** mRNA expression of HO-1 in the spleen from control and *F. hepatica*-infected mice at 3 wpi. The figures represent the results of three independent experiments (±SEM, indicated by error bars). Mice were analyzed individually: control mice *n* = 12 and infected mice *n* = 17. Asterisks indicate statistically significant differences (***p < 0.001). The bar represents 100 µm.

We first evaluated whether HO-1 was expressed in *F. hepatica*-infected animals. To this end, mice were infected with 10 metacercariae and after 3 wpi, livers, spleens, and PECs were removed and HO-1 expression was analyzed by qRT-PCR, microscopy, and flow cytometry. Livers from infected mice expressed high levels of HO-1, both at the mRNA (Figure 1A) and protein levels (Figure 1B). Indeed, a 25-fold increase in the transcript levels was determined by qRT-PCR with respect to non-infected animals (Figure 1A). HO-1 expression was found both in the leukocyte infiltrates and the liver parenchyma (Figure 1B), while undetectable levels of HO-1 were found in control livers from naive mice (Figure 1B). HO-1 gene expression was also induced in PECs, revealing, similar to liver, a 25-fold increased in PECs from infected animals, comparing to control mice (Figure 1C). Moreover, HO-1^+^ cells were detected in the peritoneum both by flow cytometry and microscopy (Figures 1D–F). On the contrary, we failed to detect an increase in HO-1 transcript levels and protein expression by flow cytometry in spleens from infected- with respect to control animals (Figure S1 in Supplementary Material).

**Figure 2:**
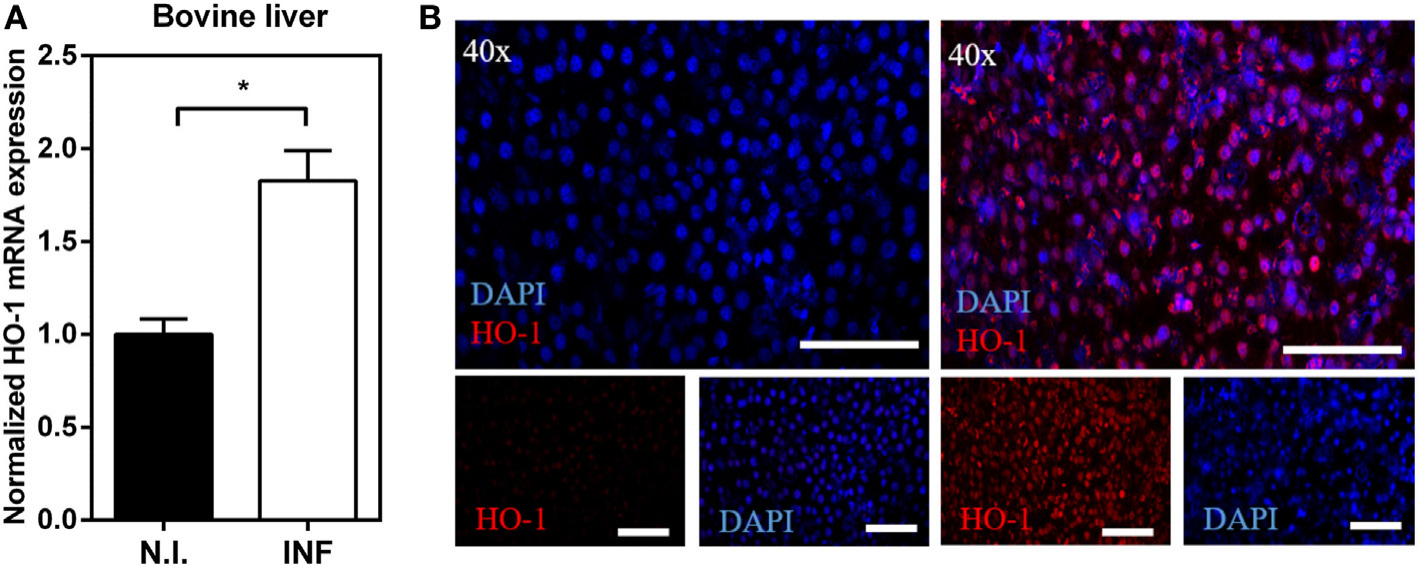
Upregulation of heme-oxygenase-1 (HO-1) expression in liver from *Fasciola hepatica*-infected bovines. **(A)** mRNA expression of HO-1 in the liver from *F. hepatica*-infected (*n* = 7) and control (*n* = 5) animals. **(B)** HO-1 expression in the liver from infected and control animals by confocal microscopy. Asterisks indicate statistically significant differences (*p < 0.05). The bar represents 100 µm.

The gene expression of HO-1 was also investigated in bovine livers (Figure 2) revealing an increase of HO-1 mRNA levels in livers from infected bovine with respect to non-infected animals (Figure 2A). This increase in HO-1 gene expression was confirmed at the protein level by microscopy (Figure 2B). HO-1 was expressed both in the hepatocytes (larger cells) and the infiltrated leukocytes (smaller cells) in livers from infected mice (Figure 2B). Altogether, these results indicate that HO-1 expression increases both in liver and PEC, but not in spleen, of *F. hepatica*-infected animals.

### The Pharmacological Inhibition or Induction of HO-1 Affects Clinical Signs Associated to *F. hepatica* Infection

**Figure 3:**
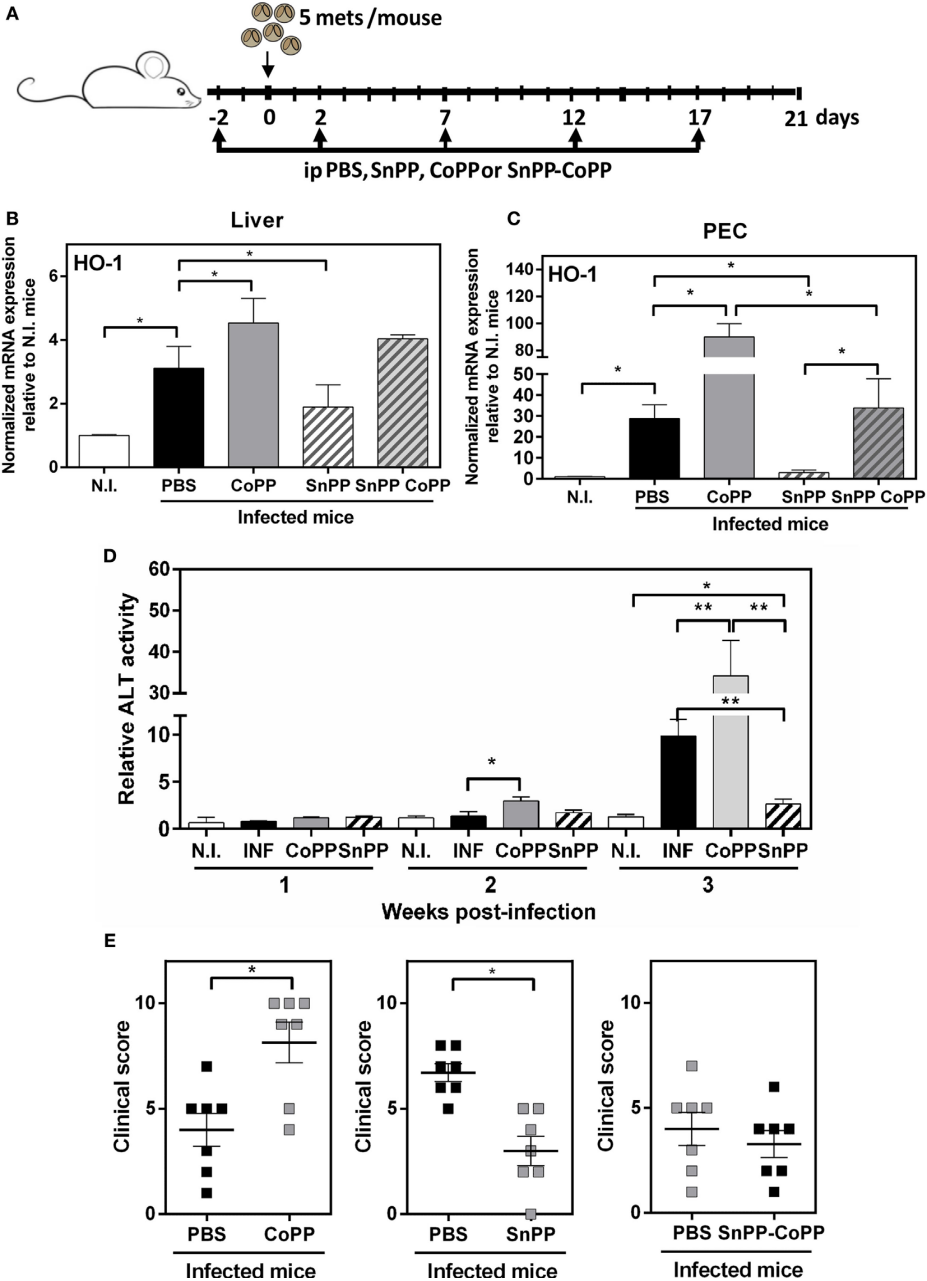
Pharmacological induction and inhibition of heme-oxygenase-1 (HO-1) alters the clinical signs associated with *Fasciola hepatica* infection. **(A)** Treatment of infected mice with CoPP, SnPP, SnPP/CoPP, or PBS (control). **(B)** mRNA expression of HO-1 in liver from CoPP-, SnPP-, and SnPP/CoPP-treated *F. hepatica*-infected mice at 3 wpi. **(C)** mRNA expression of HO-1 in peritoneal exudate cells (PECs) from CoPP-, SnPP-, and SnPP/CoPP-treated *F. hepatica*-infected mice at 3 wpi. **(D)** Alanine aminotransferase (ALT) activity was measured in sera from CoPP- and SnPP-treated infected and control mice. **(E)** Clinical score of CoPP- and SnPP-treated *F. hepatica*-infected mice at 3 wpi, according to Table 1. The figures represent the results of three independent experiments (±SEM, indicated by error bars). Mice were analyzed individually: CoPP (*n* = 7), SnPP (*n* = 7), SnPP/CoPP (*n* = 7), or PBS (*n* = 7). Asterisks indicate statistically significant differences (*p < 0.05, **p < 0.01).

The use of pharmacological agents and genetic probes to manipulate HO-1 has been widely used as a tool to explore the role of HO-1 in infections and other pathological systems, as well as its immune regulatory properties. Thus, we investigated whether the pharmacological induction or inhibition of HO-1, using cobalt (CoPP) and tin (SnPP) protoporphyrin IX, respectively, increased or ameliorated the clinical signs associated with by *F. hepatica* infection. The treatment consisted of five i.p. administrations of CoPP or SnPP at days −2, 2, 5, 12, and 17, with infection at day 0 (Figure 3A). Importantly, CoPP administration lead to a significant increase of HO-1 transcript levels, while SnPP administration did not change the HO-1 gene expression (Figures S2A,B in Supplementary Material) Clinical signs were determined by two different read outs: (i) hepatic damage followed by ALT activity in serum, a common marker to detect hepatic dysfunction (30), and (ii) general state of the animal by a defined clinical score (Figure 3). The clinical score was defined according the parameters described in Table 1. First, we evaluated the HO-1 transcript levels in livers and PECs from treated mice at 2 wpi, time were the highest differences in HO-1 expression were determined. Infected mice expressed high transcript levels of HO-1, both in liver (Figure 3B) and PEC (Figure 3C). Furthermore, when infected mice were treated with CoPP, they presented higher HO-1 transcript levels than infected mice in both biological samples, while SnPP-treatment dramatically reduced the gene expression of HO-1 in infected mice, both in liver (Figure 3B) and PEC (Figure 3C). Of note, when infected mice were treated with simultaneous administration of CoPP and SnPP, the HO-1 transcript levels both in PEC and liver were similar to those found for infected control mice (Figures 3B,C).

Importantly, the expression of HO-1 correlated with the ALT activity levels found in sera. Indeed, CoPP-treated infected mice presented higher ALT activity levels in serum at 2 and 3 wpi (Figure 3D). On the contrary, SnPP-treated infected mice, had a remarkable decrease in ALT activity levels at 3 wpi with levels comparable to those of non-infected mice, although they were slightly increased (Figure 3D). The hepatic damage determined as ALT activity in serum found in CoPP-treated infected mice correlated with other clinical signs, such as hemorrhage, splenomegaly and increase in ascites and cells in the peritoneum (Figure 3E). In contrast, SnPP-treated infected mice, presented a decreased clinical score as compared to controls. Importantly, control infected mice treated with a mix of SnPP and CoPP, presented the same clinical score as infected mice not treated with protoporfirins (Figure 3E). Importantly, non-infected mice treated with CoPP did not show either liver damage, changes in ALT activity in sera nor any clinical symptom related to the infection with respect to non-treated mice (Figure S2C,D in Supplementary Material). These results suggest that an increase of HO-1 expression augments the susceptibility of *F. hepatica* infection, while a decrease in this enzyme provides mice resistance to the infection.

**Figure 4:**
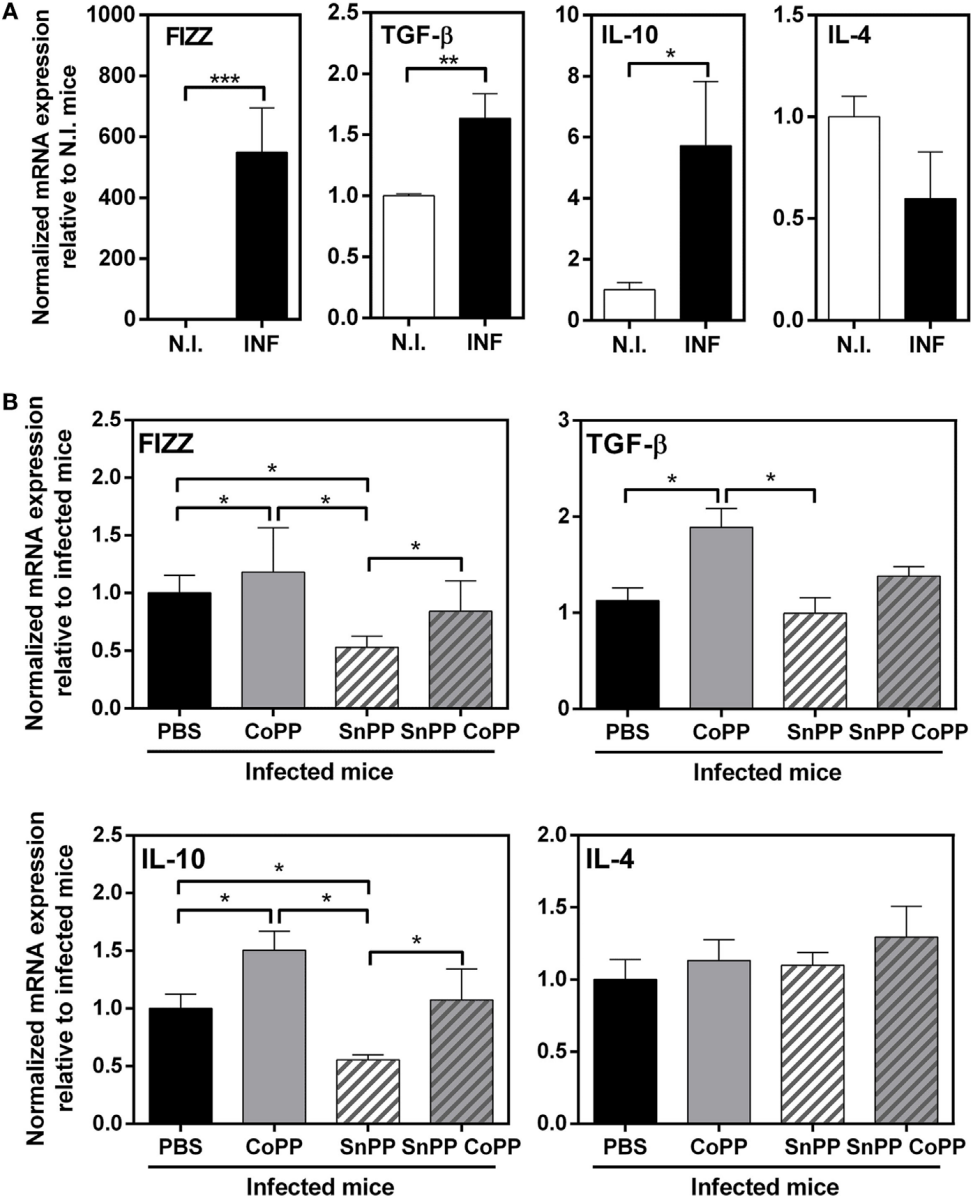
Pharmacological induction of heme-oxygenase-1 expression correlates with IL-10, TGFβ, and FIZZ-1 transcript levels in liver of infected animals. **(A)** mRNA expression of IL-4, IL-10, TGFβ, and FIZZ-1 in the liver from control and *F. hepatica*-infected mice at 2 wpi. **(B)** mRNA expression of IL-4, IL-10, TGFβ, and FIZZ-1 in the liver from CoPP-, SnPP- and SnPP/CoPP-treated *F. hepatica*-infected mice at 2 wpi. The figures represent the results of three independent experiments (±SEM, indicated by error bars). Mice were analyzed individually: CoPP (*n* = 7), SnPP (*n* = 7), SnPP/CoPP (*n* = 7), or PBS (*n* = 7). Asterisks indicate statistically significant differences (*p < 0.05, **p < 0.01, ***p < 0.001).

It has been reported that HO-1 regulates the expression of multiple cytokines, and has essentially anti-inflammatory properties (26, 31–34). In order to further study the immune response induced in the group of mice treated with protoporphyrins, we evaluated the transcript levels of a panel of Th2/regulatory molecules that are highly expressed during *F hepatica* infection: FIZZ-1, IL-4, IL-10, and TGFβ. Indeed, at 2 wpi, livers from infected mice expressed high transcript levels of IL-10, TGFβ and FIZZ-1 (Figure 4A). Interestingly, IL-10 and TGFβ transcript levels were even higher in CoPP-treated infected mice, than control infected mice (Figure 4B). Moreover, SnPP-treated infected mice presented lower mRNA levels of IL-10 and FIZZ-1 than infected control mice (Figure 4B). Consistent with these results, simultaneous treatment with CoPP and SnPP did not induce any change in the mRNA levels of these molecules with respect to control infected mice (Figure 4B). Of note, IL-4 gene expression in liver was not modified either with *F. hepatica* infection nor the treatment with metal protoporphyrins.

**Figure 5:**
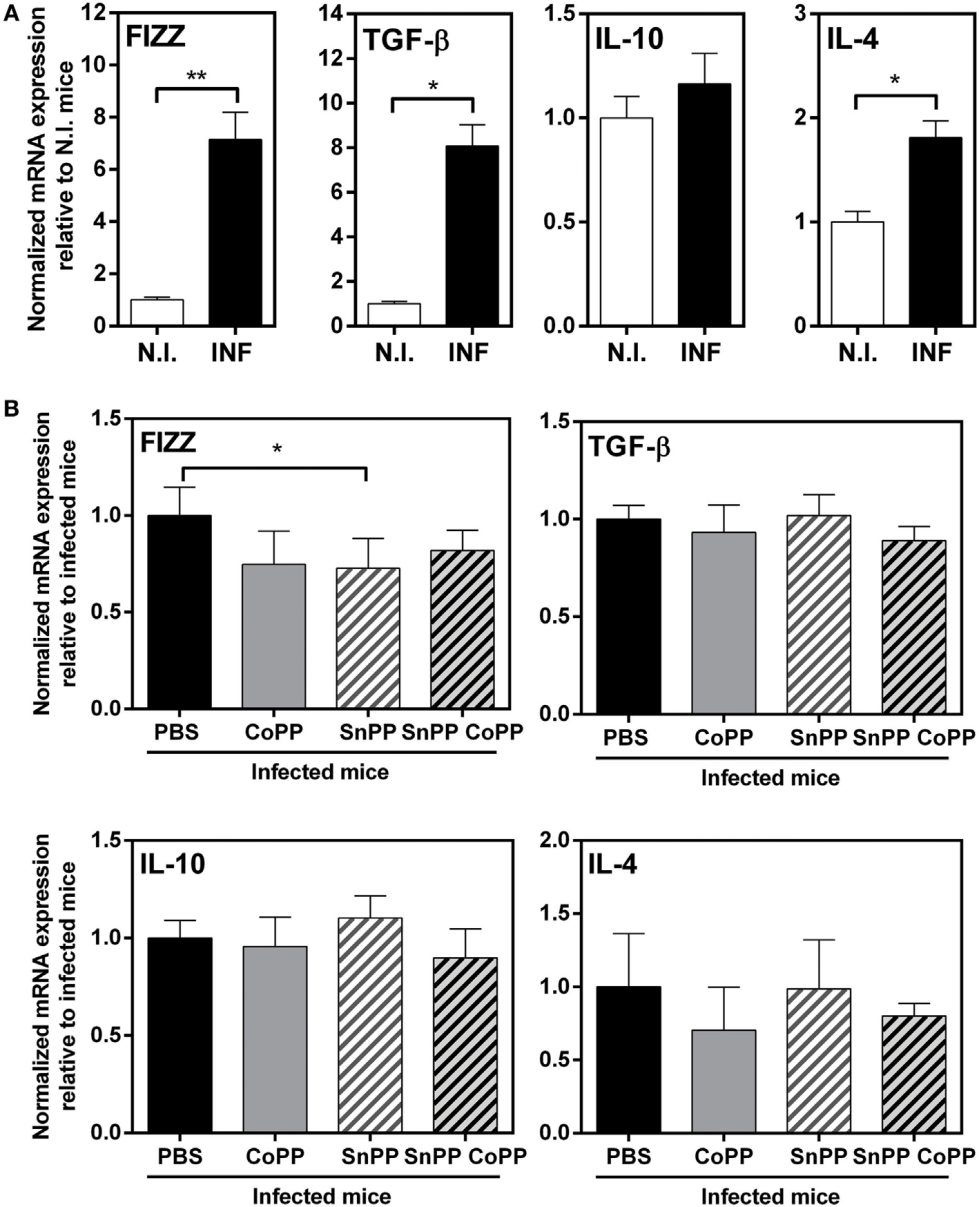
IL-4, IL-10, TGFβ, and FIZZ-1 mRNA levels in peritoneal exudate cells (PECs) from CoPP-, SnPP-, and SnPP/CoPP-infected animals. **(A)** mRNA expression of IL-4, IL-10, TGFβ, and FIZZ-1 in PEC from control and *Fasciola hepatica*-infected mice at 2 wpi. **(B)** mRNA expression of IL-4, IL-10, TGFβ, and FIZZ-1 in PEC from CoPP-, SnPP- and SnPP/CoPP-treated *F. hepatica*-infected mice at 2 wpi. The figures represent the results of three independent experiments (±SEM, indicated by error bars). Mice were analyzed individually: CoPP (*n* = 7), SnPP (*n* = 7), SnPP/CoPP (*n* = 7), or PBS (*n* = 7). Asterisks indicate statistically significant differences (*p < 0.05, **p < 0.01).

Cells from the peritoneum of infected mice, on the other hand, expressed higher transcript levels of TGFβ, IL-4 and FIZZ-1 (Figure 5A), but not IL-10 as shown in liver. Surprisingly, PEC from CoPP- or SnPP-treated mice did not present any change in the expression of either TGFβ, IL-4, FIZZ-1 or IL-10, except for FIZZ-1 which was slightly decreased in SnPP-treated infected mice (Figure 5B), consistent with lower hepatic damage and clinical score.

In summary, these results show that the induction of HO-1 is associated with higher levels of the immunoregulatory molecules IL-10 and TGFβ and the reparatory molecule FIZZ-1 in liver, while the inhibition of HO-1correlated with lower expression of TGFβ and FIZZ-1 in the liver of infected animals. However, although infected animals presented increased levels of TGFβ, IL-4 and FIZZ-1 on peritoneal cells, we could not find significant changes associated with the modulation of HO-1. Considering that the peritoneum is essential for *F. hepatica* juvenile maturation, we studied in further detail peritoneal cells and the expression of HO-1.

### HO-1 Is Induced in Two Different Peritoneal F4/80^+^ Cell Populations

**Figure 6:**
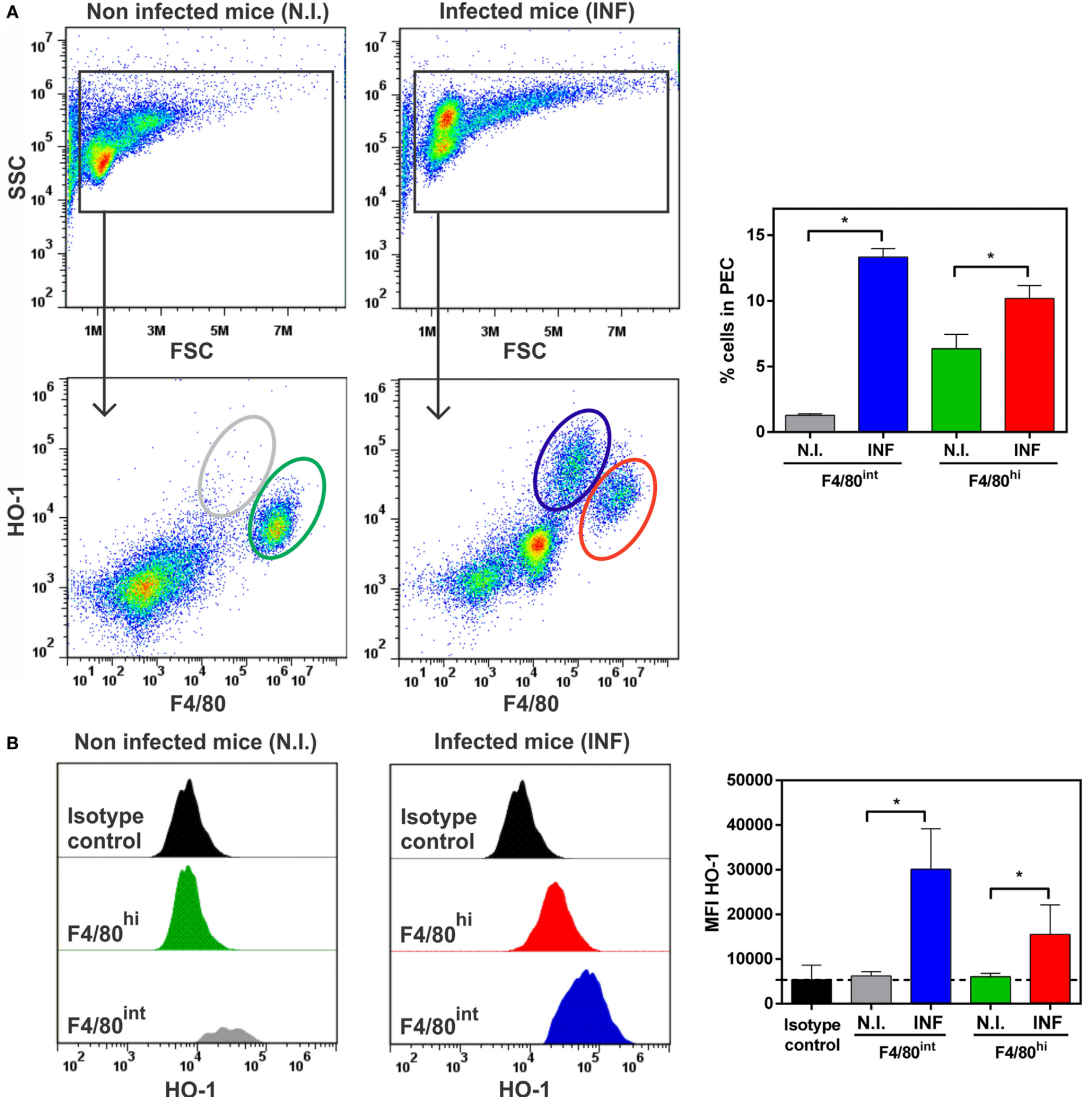
HO-1^+^ cells recruited in peritoneal exudate cell (PEC) during *Fasciola hepatica* infection are F4/80^+^. **(A)** HO-1^+^ and F4/80^+^ cells in PEC from control and infected mice at 3 wpi by flow cytometry. Percentage of HO-1^hi^ F4/80^int^ and HO-1^low^ F4/80^hi^ cells in the peritoneum from control and infected mice at 3 wpi. **(B)** heme-oxygenase-1 (HO-1) expression in different cell populations from PEC from control and infected mice at 3 wpi. MFI (Mean Fluorescence Intensity) of HO-1 in HO-1^hi^ F4/80^int^ and HO-1^low^ F4/80^hi^ cells from PECs by flow cytometry. The figures represent the results from at least three independent experiments (±SEM, indicated by error bars). Mice were analyzed individually: CoPP (*n* = 7), SnPP (*n* = 7), SnPP/CoPP (*n* = 7), or PBS (*n* = 7). Asterisks indicate statistically significant differences (*p < 0.05).

Considering previous reports demonstrating that: (i) *F. hepatica*-infected mice express high levels of IL-10 (8, 35), (ii) HO-1 expression is related to IL-10 signaling and viceversa (36, 37), (iii) alternatively activated macrophages are associated with *F. hepatica* infection (9, 38, 39), and (iv) HO-1 is highly expressed by M2 macrophages (40), we sought to evaluate whether the HO-1^+^ cells identified in *F. hepatica*-infected mice expressed the molecule F4/80, traditionally used to identify macrophages. As seen in Figure 6A, HO-1^+^ cells from PECs from infected animals expressed this surface marker. However, two HO-1^+^ populations were identified according to the expression of HO-1 and F4/80: HO-1^int^ F4/80^hi^ and HO-1^hi^ F4/80^int^, which significantly augmented upon infection (Figure 6A). Interestingly, although the F4/80^hi^ cell population was also detected in PECs from control mice (Figure 6A), the expression of HO-1 was induced upon infection (Figure 6B). On the contrary, the HO-1^hi^ F4/80^int^ cell population was absent in control mice (Figure 6A) and expressed higher HO-1 levels than HO-1^int^ F4/80^hi^ cells from infected mice (Figure 6B). Of note, the F4/80^low^ population identified in infected mice were Siglec-F^+^ (could correspond to eosinophils) and did not express HO-1 as determined by the corresponding isotype staining (Figure S3 in Supplementary Material).

**Figure 7:**
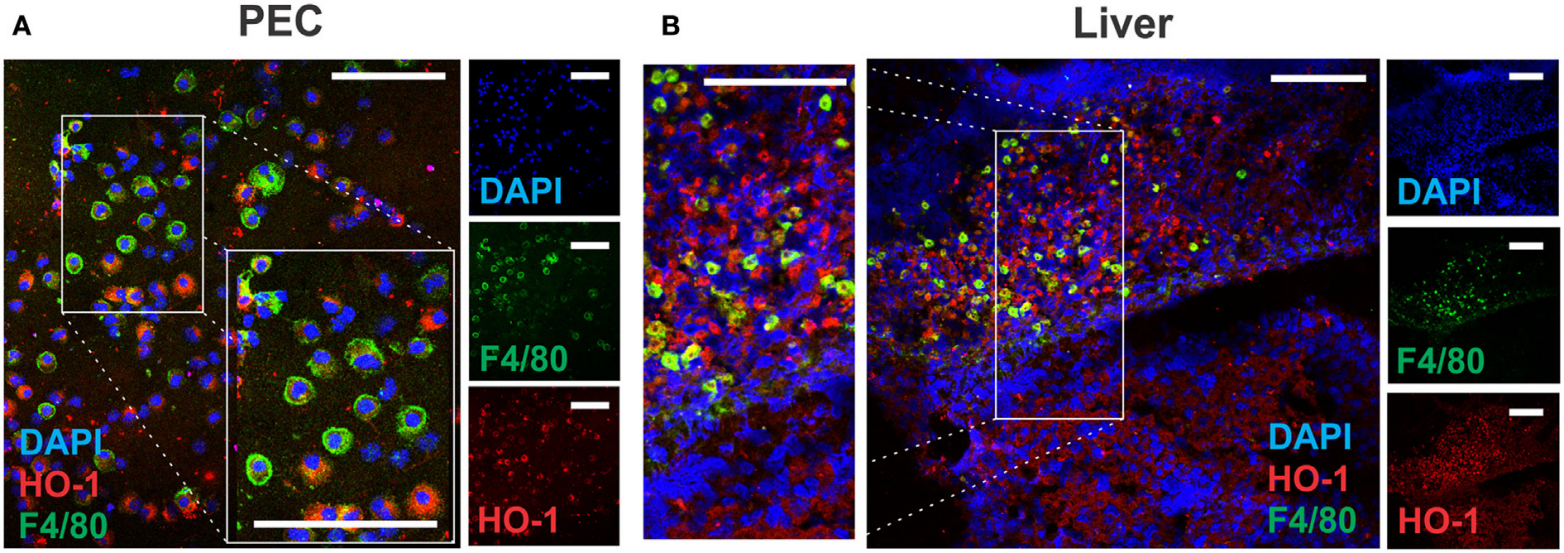
Identification of HO-1^+^ cells in peritoneal exudate cell (PEC) and liver by confocal microscopy. Heme-oxygenase-1 (HO-1) and F4/80 expression in PEC **(A)** and liver **(B)** from control (*n* = 5) and infected mice (*n* = 5) at 3 wpi by confocal microscopy. DAPI was used to stain cell nucleus. The bar represents 100 µm. The figures represent the results from at least three independent experiments.

The presence of F4/80^+^ cells in PECs expressing different levels of this surface marker was also confirmed by microscopy, revealing co-localization with HO-1 (Figure 7A). Furthermore, F4/80^+^ HO-1^+^ cells were also identified in the leukocyte infiltrate present in livers from infected mice (Figure 7B), while these cells were undetected in control livers (data not shown). HO-1 was also expressed by F4/80^-^ hepatocytes (Figure 7B) as mentioned earlier (Figure 1B).

### HO-1^int^ F4/80^hi^ and HO-1^hi^ F4/80^int^ Cells from Infected Mice Have Different Phenotype

**Figure 8:**
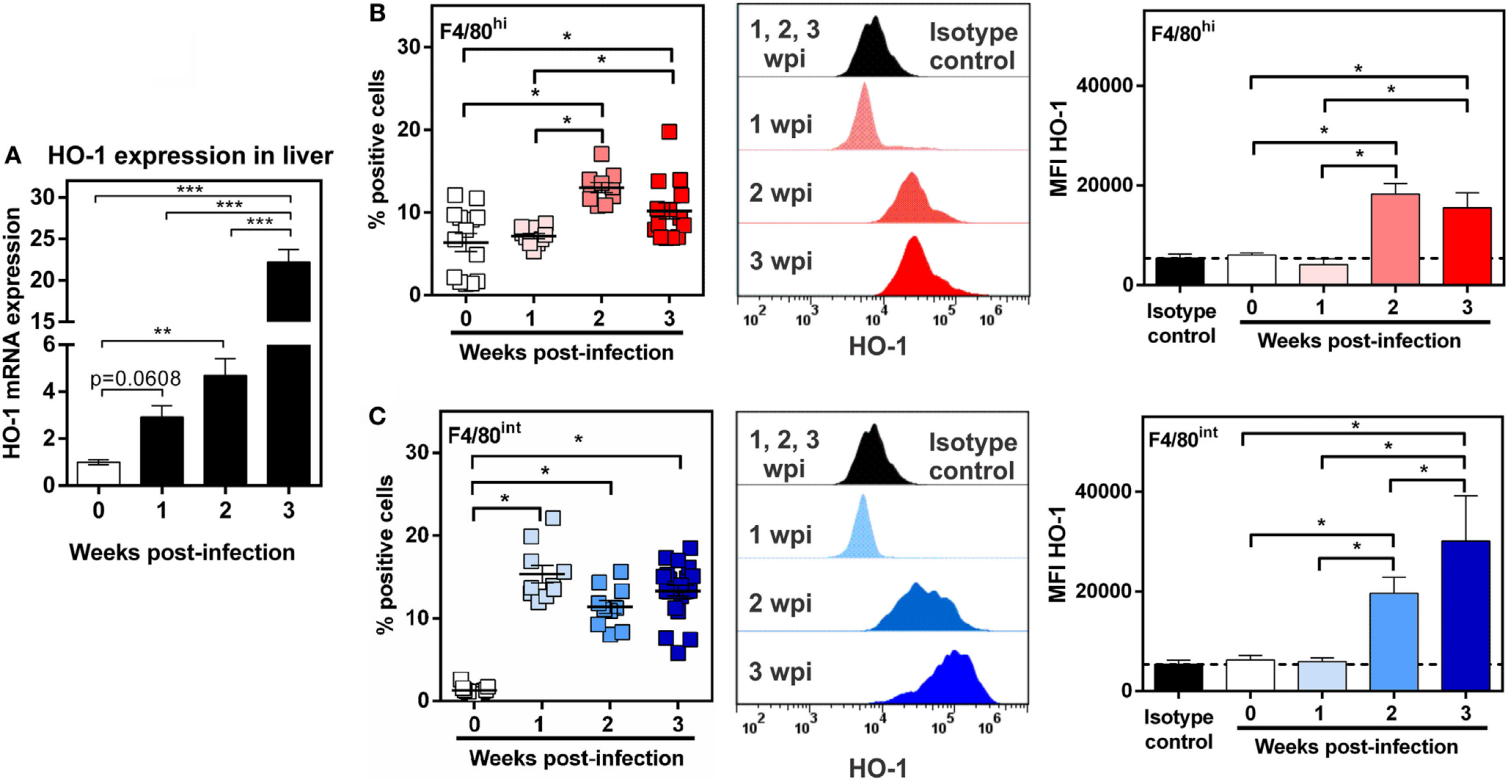
Identification of HO-1^+^ cells during *Fasciola hepatica-*infection. **(A)** mRNA expression of heme-oxygenase-1 (HO-1) in the liver from control (0 wpi) and infected mice (1, 2, and 3 wpi). **(B)** HO-1^+^ F4/80^hi^ cells in peritoneal exudate cell (PEC) from control (0 wpi) and infected mice (1, 2, and 3 wpi) by flow cytometry. On the right the HO-1 expression as the MFI of HO-1^+^ F4/80^hi^ cells is shown. **(C)** HO-1^+^ F4/80^int^ cells in PEC from control (0 wpi) and infected mice (1, 2, and 3 wpi) by flow cytometry. On the right, the HO-1 expression as the MFI of HO-1^+^ F4/80^int^ cells is shown. Mice were analyzed individually: control (*n* = 12), 1 wpi (*n* = 10), 2 wpi (*n* = 10) or 3 wpi (*n* = 16). Asterisks indicate statistically significant differences (*p < 0.05, **p < 0.01, ***p < 0.001).

In order to further characterize HO-1^+^ cells, we evaluated both populations and HO-1 expression during the process of infection. To this end, PECs and livers were collected at 1, 2, and 3 wpi. Interestingly HO-1 transcript levels augmented progressively with the course of infection (Figure 8A). PECs from infected and control mice were labeled and analyzed by flow cytometry in order to identify both HO-1^+^ cell populations, HO-1^hi^ F4/80^int^ and HO-1^int^ F4/80^hi^, and compare them with PECs from control mice. HO-1^int^ F4/80^hi^ cells were already present in control mice and its number doubled from the second week post infection (Figure 8B), time in which they presented increased levels of HO-1 expression (Figure 8B). On the other hand, HO-1^hi^ F4/80^int^ cells in PECs were detected as soon as 1 wpi, and remained constant up to 3 wpi (Figure 8C). The expression of HO-1 by these cells was induced from 2 wpi (Figure 8C). Both the cell number and the HO-1 expression by HO-1^int^ F4/80^hi^ cells remained constant or increased after 2 wpi (Figure 8C).

**Figure 9:**
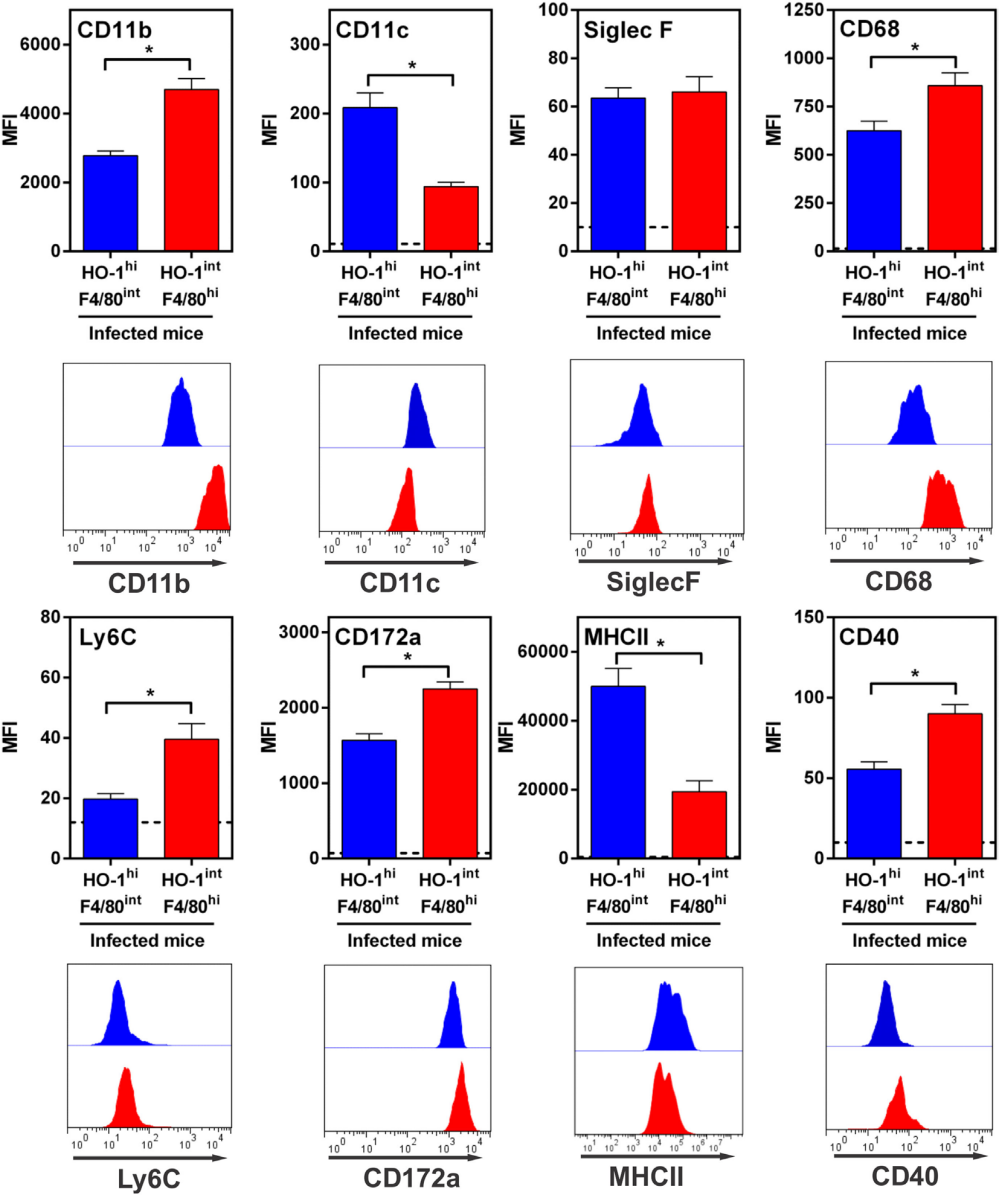
Immunophenotyping of HO-1^hi^ F4/80^int^ and HO-1^int^ F4/80^hi^ in the peritoneum of *Fasciola hepatica*-infected animals. HO-1^hi^ F4/80^int^ cells (blue), HO-1^int^ F4/80^hi^ cells (red) from peritoneal exudate cells of infected mice at 3 wpi were evaluated for the expression of different cell markers by flow cytometry. Cells from the peritoneal cavity from infected mice were stained with CD11b- CD11c-, MHCII, CD40, SIRPα-, CD68-, Ly6C-, or Siglec-F- specific antibodies and evaluated by flow cytometry. A representative figure of three independent experiments is shown. Mice were analyzed individually: control mice *n* = 5 and *F. hepatica*-infected mice *n* = 5. Asterisks indicate statistically significant differences (*p < 0.05).

We then investigated the phenotype of HO-1^+^ cells by evaluating the expression of different molecules by flow cytometry and compared them to F4/80^+^ cells found in naïve mice (Figure 9; Figure S4 in Supplementary Material). HO-1^int^ F4/80^hi^ and HO-1^hi^ F4/80^int^ cells expressed CD11b, CD68 and CD172a (SIRPα, Figure 9), all molecules that are expressed by DCs or macrophages (41). However, HO-1^int^ F4/80^hi^ cells expressed higher levels of CD11b, CD68 and CD172a than HO-1^hi^ F4/80^int^ cells. Furthermore, HO-1^hi^ F4/80^int^ cells expressed CD11c while HO-1^int^ F4/80^hi^ expressed Ly6C (Figure 9). Finally, HO-1^hi^ F4/80^int^ cells expressed higher levels of MHC class II but lower expression of CD40 than HO-1^int^ F4/80^hi^ cells. Of note, both cells populations expressed very low levels of Siglec-F (Figure 9). Interestingly, the phenotype described for HO-1^int^ F4/80^hi^ cells resembled to that of peritoneal macrophages from naïve mice (Figure S4 in Supplementary Material). Altogether, these results suggest that HO-1^hi^ F4/80^int^ cells could constitute DCs while HO-1^int^ F4/80^hi^ cells would correspond to monocytes or macrophages.

**Figure 10:**
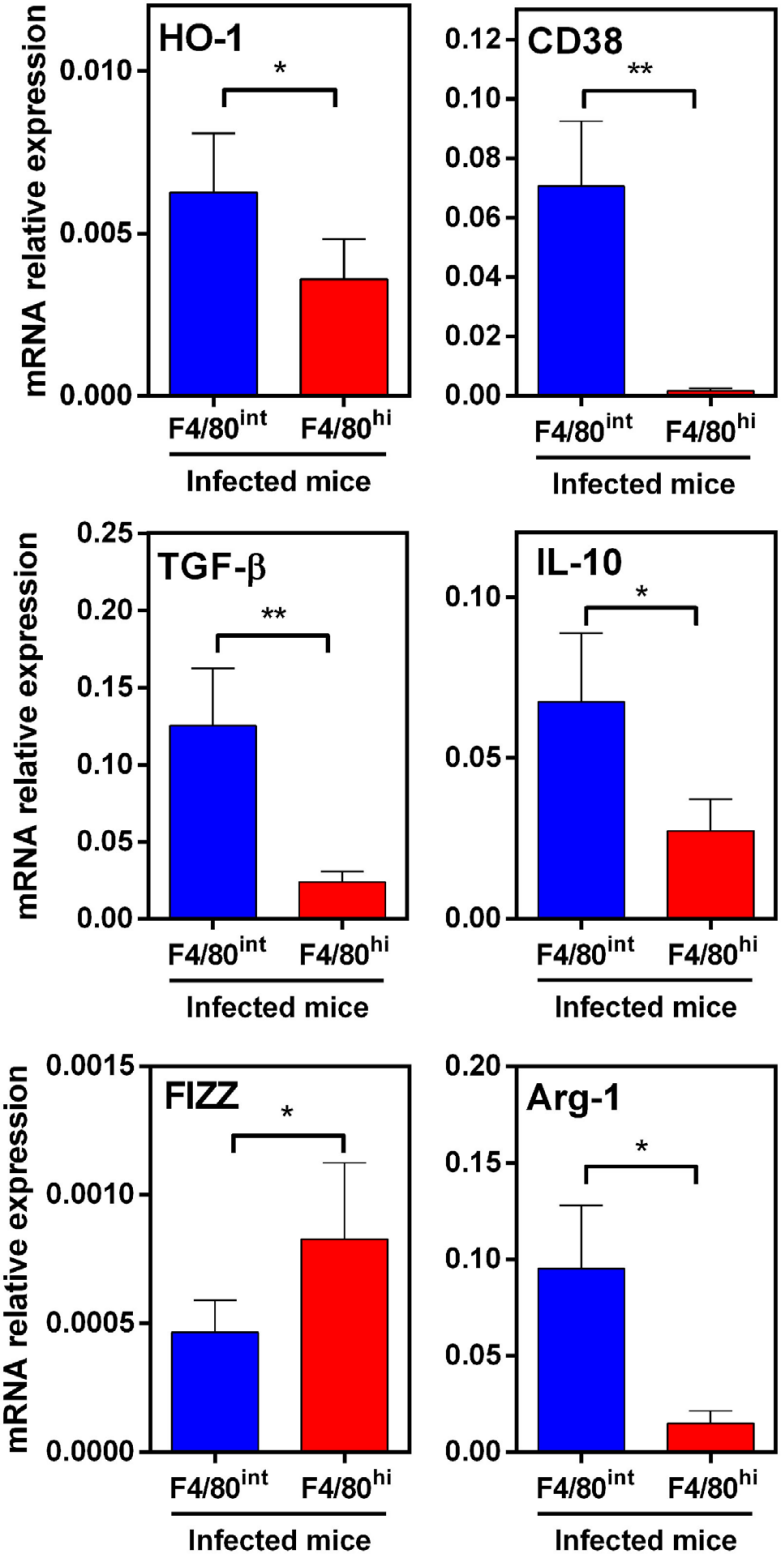
Expression of immunoregulatory molecules in HO-1^hi^ F4/80^int^ and HO-1^int^ F4/80^hi^ in peritoneal exudate cells (PECs) from *Fasciola hepatica*-infected animals. HO-1^hi^ F4/80^int^ cells (blue), HO-1^int^ F4/80^hi^ cells (red) from PECs of infected mice at 3 wpi were first sorted. Then, the expression of heme-oxygenase-1 (HO-1), TGFβ, IL-10, FIZZ-1, Arg-1, and CD38 was evaluated by qRT-PCR. Gene expression relative to GAPDH transcript levels is shown. Mice were analyzed individually: control mice *n* = 5 and *F. hepatica*-infected mice *n* = 5. Asterisks indicate statistically significant differences (*p < 0.05, **p < 0.01).

To further characterize these cells, we sorted them by flow cytometry and analyzed the gene expression of other molecules by qRT-PCR. In agreement with flow cytometry analyses, HO-1^hi^ F4/80^int^ cells expressed higher transcript levels of HO-1 than HO-1^int^ F4/80^hi^ cells (Figure 10). Interestingly, both cells populations were very different in the set of expressed genes. Indeed, HO-1^hi^ F4/80^int^ cells expressed CD38 and Arg-1, while HO-1^int^ F4/80^hi^ cells did not. On the contrary, HO-1^int^ F4/80^hi^ cells expressed FIZZ-1 and IL-10. Finally, HO-1^hi^ F4/80^int^ cells expressed TGFβ and IL-10 (Figure 10). According to the expression of these markers, these results suggest that HO-1^hi^ F4/80^int^ cells correspond to regulatory or tolerogenic DCs, while HO-1^int^ F4/80^hi^ cells could constitute alternatively activated macrophages.

## Discussion

In this work, we show that HO-1 is a key immunoregulatory molecule during *F. hepatica* infection and that promotes infection and liver damage. The role of HO-1 in infections by intracellular pathogens has been previously approached, demonstrating an upregulation of HO-1 mRNA and/or protein expression in response to viral (25), bacterial (23, 41–46), or protozoan parasitic (18, 19, 47) infections. Furthermore, overexpression or induction of HO-1 promotes persistence of other infectious agents, such as *Leishmania chagasi* and *Plasmodium* liver infection (18, 47). However, to our knowledge, this is the first report demonstrating the role of HO-1 in favoring a helminth infection.

The involvement of HO-1 in the anti-inflammatory immune response in *F. hepatica*-infected mice was confirmed using pharmacological approaches. We show that the pharmacological induction of HO-1 promoted clinical signs associated with *F. hepatica* infection, and it was correlated with an increase of IL-10 and TGFβ in liver, indicating that the induction of HO-1 is associated with the upregulation of these two immunoregulatory cytokines. The fact that the use of the enzymatic inhibitor of HO-1 SnPP significantly decreased the levels of IL-10, TGFβ, and FIZZ-1 in liver, even to lower levels to control infected mice (for IL-10 and FIZZ-1) strongly suggests that HO-1 is involved in the upregulation of IL-10, promoting parasite survival, and hence liver damage that leads to the upregulation of FIZZ-1 indicating liver fibrosis. Indeed, several studies have demonstrated that HO-1 mediates the anti-inflammatory effect of IL-10 (37, 48) showing that the use of competitive inhibitors or the knock down expression of HO-1 abrogated the suppressive effect of IL-10. In our model, this hypothesis is in agreement with the results obtained with the simultaneous administration of CoPP and SnPP, obtaining similar clinical signs and IL-10, TGFβ, and FIZZ-1 levels as non-treated mice. Further studies are needed to define which of the heme degradation products following the action of HO-1 activity iron, biliverdin, or CO, are responsible for these actions, as has been previously reported for other pathogens (23). Alternatively, cytokine induction may be due to direct interaction of HO-1 with other host molecules. Interestingly, HO-1 gene expression is regulated at the transcriptional level, by several transcriptional factors including activator protein-1 (49, 50), nuclear factor erythroid 2-related factor-2 (NRF2), nuclear factor-kappa B (50, 51), among others. Also, HO-1 expression is regulated by signaling cascades such as mitogen-activated protein kinase and phosphatidylinositol 3-kinase/Akt (49, 52). In our model, the identification of the molecular mechanisms that lead to HO-1 upregulation in *F. hepatica*-infected animals will eventually contribute to the development of molecular strategies to control the infection.

Apart from its immunoregulatory properties, HO-1 also plays a significant role in inhibiting oxidant-induced injury during inflammatory processes (53). In fact, an appropriate balance of the inflammatory and redox states is essential to resolve most infections and finally the infectious process (17). In this context, another possibility is that *F. hepatica* induces HO-1 expression not only to evade the host immune response, but also to inhibit oxidant production by macrophages or other cells. One immunologically relevant place in the host for *F. hepatica*, is the peritoneal cavity, where the production of oxygen or nitrogen derived molecules could limit and restrain juvenile parasites. Indeed, lower levels of liver damage have been suggested to be the consequence of effective killing of the invading parasites within the peritoneum or shortly after reaching the liver (54). In this context, *F. hepatica*-mediated HO-1 induction might support parasite survival, for instance by favoring its passage through the peritoneum to the liver.

Our data indicate that in the peritoneal cavity two different populations of antigen presenting cells express HO-1. Judged by the high expression of CD11c, CD38, MHCII and the immunoregulatory cytokines IL-10 and TGFβ, HO-1^hi^ F4/80^int^ cells could constitute tolerogenic myeloid-derived DCs (55) or regulatory DCs that potentially participate in the induction of specific regulatory or anergic T cells (8, 56). Indeed, DCs conditioned with parasite-derived molecules can induce T cell anergy (8, 14, 56, 57). It remains to be determined whether HO-1-expressing DCs can induce specific anergic or regulatory T cells in a HO-1 dependent mechanism. In contrast, HO-1^int^ F4/80^hi^ cells were characterized by the high expression of CD68, CD172a, Ly6C, CD11b, and FIZZ-1, as well as low levels of MHCII expression, indicating that they may correspond to alternatively activated macrophages (58). In this line, the alternative activation of macrophages by *F. hepatica* or its derived molecules has been previously described (10, 39, 59, 60). Macrophages play a central role in innate immune responses toward both extracellular and intracellular pathogens, particularly through the formation of reactive oxygen/nitrogen species (RO/NS) (61, 62). Indeed, oxidative stress can kill *F. hepatica* flukes by a mechanism that may involve oxidation of proteins or lipids from parasite tegument since peroxyntrite or superoxide radicals significantly diminished parasite viability *in vitro* (54, 63). Moreover, RO/NS can effectively target extracellular pathogens through the formation of extracellular traps (61). Taking into account that HO-1 in macrophages limits the production of reactive species (34) and induces IL-10 producing anti-inflammatory macrophages (64) and that *F. hepatica* favors the alternative activation of macrophages (65, 66), it is likely that HO-1^+^ macrophages at early stages allow *F. hepatica* survival in the peritoneum through ineffective free radical production.

Finally, the role of HO-1 in favoring *F. hepatica* infection in the natural host (e.g., livestock, human) remains unknown. Although we show preliminary data demonstrating an increase in HO-1 expression in livers from naturally infected cattle, further studies are necessary to determine whether HO-1 expression correlates with a certain stage of the infection or if participates in the immunoregulatory or anti-oxidant mechanisms during the infection in these hosts.

In conclusion, HO-1 overexpression benefits *F. hepatica* infection increasing clinical signs and liver damage. Upregulation of HO-1 leads to an increase of IL-10 which could promote and benefit parasite transport from the peritoneum to the liver. On the other hand, an enzymatic inhibitor of HO-1 provided mice with resistance to infection, decreasing IL-10 and FIZZ-1 transcript levels in liver. Although the mechanisms by which HO-1^+^ DCs or macrophages regulate the expression of IL-10 or oxidative responses during *F. hepatica* infection remain to be elucidated, targeting HO-1 to control fasciolosis could constitute an interesting alternative strategy to drugs or vaccines against fasciolosis.

## Ethics Statement

Mouse experiments were carried out in accordance with strict guidelines from the National Committee on Animal Research (Comisión Nacional de Experimentación Animal, CNEA, National Law 18.611, Uruguay) according to the international statements on animal use in biomedical research from the Pan American Health Organization (PAHO) and World Health Organization (WHO). The protocol was approved by the Uruguayan Committee on Animal Research. Cow’s livers were collected during the routine work of a local abattoir (Frigorífico Carrasco) in Montevideo (Uruguay). All procedures involving animals were approved by the Universidad de la República’s Committee on Animal Research (Comisión Honoraria de Experimentación Animal, CHEA Protocol Number: 070153-000180-16).

## Author Contributions

PC performed the experiments, analyzed data, and contributed with manuscript revision. ER, VC and SF contributed with mouse infections and flow cytometry experiments. VN and NB participated in obtention of flukes and extract preparation and detoxification. CR contributed with reactifs and participated in real-time RT-PCR experiments. IA contributed with reactifs, designed experiments with protoporphirn treatment and helped with manuscript revision. TF contributed to supervision and design of all experiments shown in this paper, analyzed data, and prepared the manuscript.

## Conflict of Interest Statement

The authors declare that the research was conducted in the absence of any commercial or financial relationships that could be construed as a potential conflict of interest.
