# Supplementary material for: Heme-Oxygenase-1 Expression Contributes to the Immunoregulation Induced by Fasciola hepatica and Promotes Infection
Source: Front Immunol. 2017 Jul 26;8:883. doi: 10.3389/fimmu.2017.00883 (PMC5526848; doi:10.3389/fimmu.2017.00883)
Supplement: Supplementary file 1 [file Presentation_1.pdf]

frontiers

Supplementary Material

Heme-oxygenase-1 Expression Contributes to the Immunoregulation Induced by *Fasciola hepatica* and Promotes Infection

Paula Carasi, Ernesto Rodríguez, Valeria da Costa, Sofía Frigerio, Natalie Brossard, Verónica Noya, Carlos Robello, Ignacio Anegón and Teresa Freire*

*** Corresponding author:** tfreire@fmed.edu.uy

Supplementary Figures

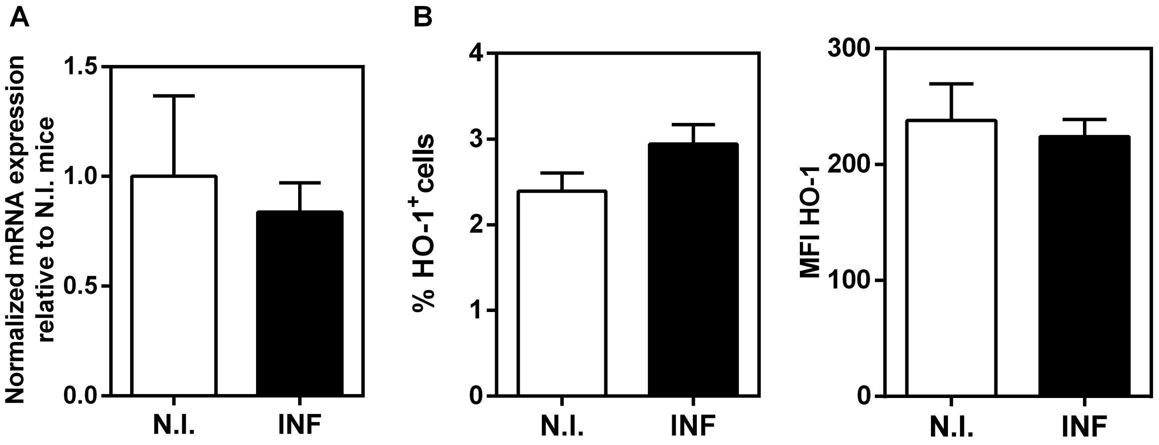

Supplementary Figure 1. HO-1 expression in spleen from infected mice. A) mRNA expression of HO-1 in spleens from control and *F. hepatica* infected mice at 3 wpi. B) Percentage and MFI (mean fluorescence intensity) of HO-1^+ cells in spleen from control and infected mice at 3 wpi by flow cytometry. The figures represent the results of three independent experiments (±SEM, indicated by error bars). Mice were analyzed individually: control mice n=12 and infected mice n=17.

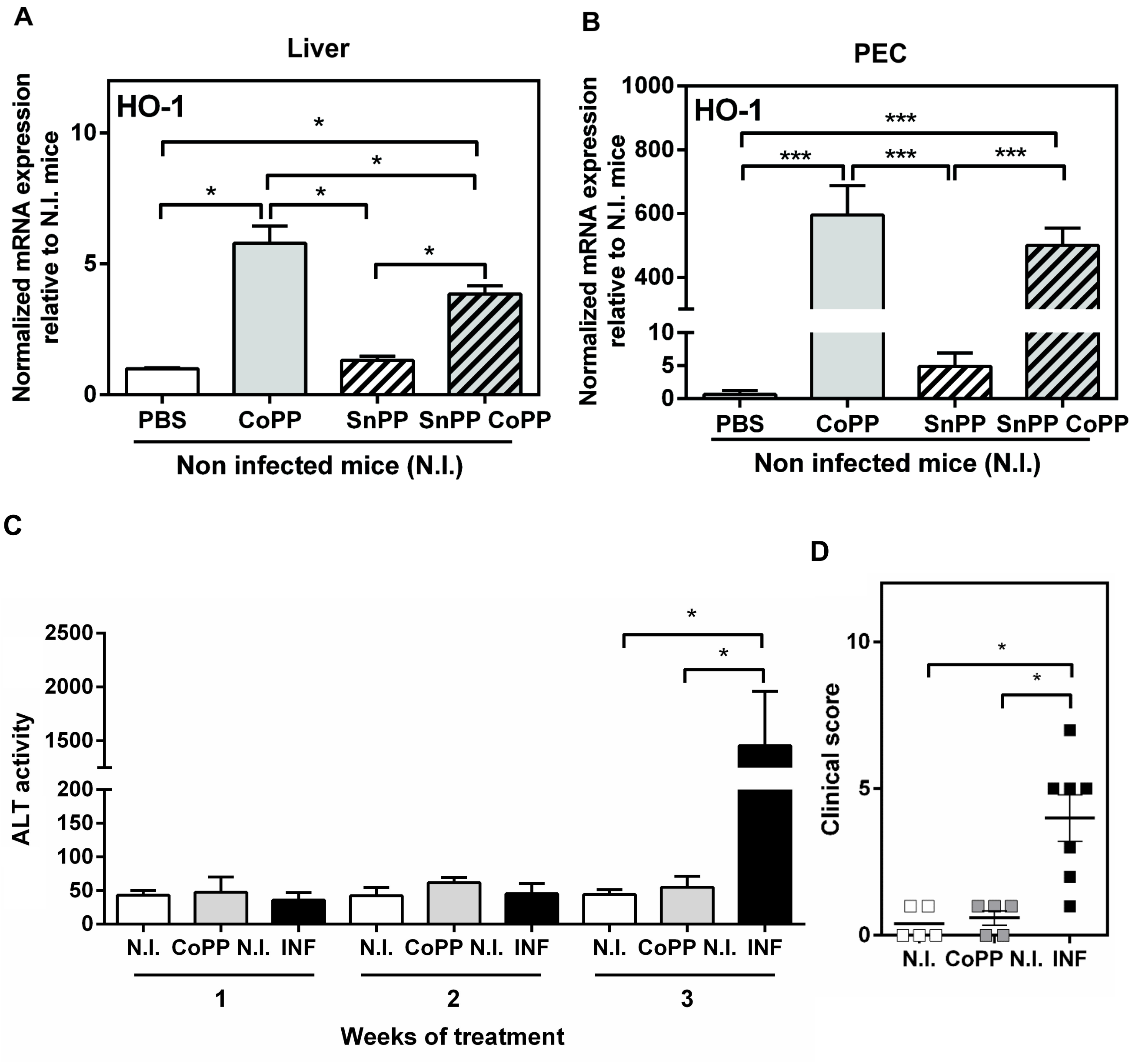

Supplementary Figure 2. Protoporphyrin treatment in control mice. HO-1 mRNA levels in liver (A) and PECs (B) from CoPP-, SnPP- and CoPP/SnPP-treated control (non infected) mice. Intraperitoneal injections of CoPP or SnPP were performed at days -2, 2, 7 and 12. C) Alanine aminotransferase (ALT) activity was measured in sera in order to evaluate liver damage in non-infected (N.I.), CoPP-treated non-infected (CoPP N.I.) and infected (INF) mice. D) Clinical score of non-infected (N.I.), CoPP-treated non-infected (CoPP N.I.) and infected (INF) mice at 3 wpi. Mice were analyzed individually: CoPP (n=5), SnPP (n=5), SnPP/CoPP (n=5), PBS (n=5) or infected (n=6). Asterisks indicate statistically significant differences (*$p < 0.05$, ***$p < 0.001$).

.

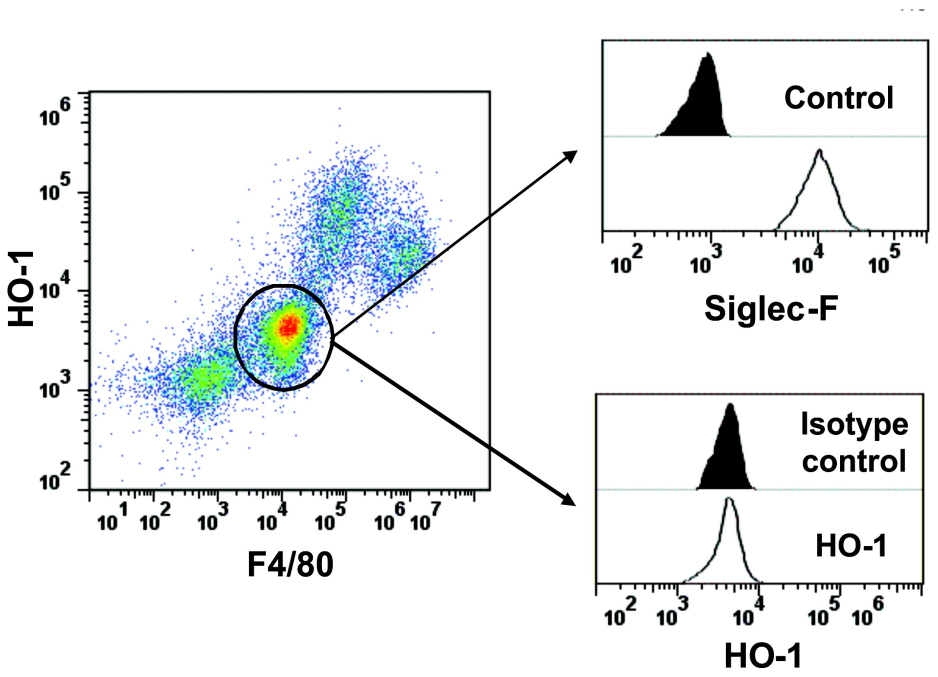

Supplementary Figure 3. F4/80^{low} cells from PECs are HO-1^{-} and Siglec-F^{-+}. Analyses by flow cytometry of the HO-1 and Siglec-F expression of F4/80^{low} cells from PECs of infected mice at 3 wpi. The figure represents the results of three independent experiments.

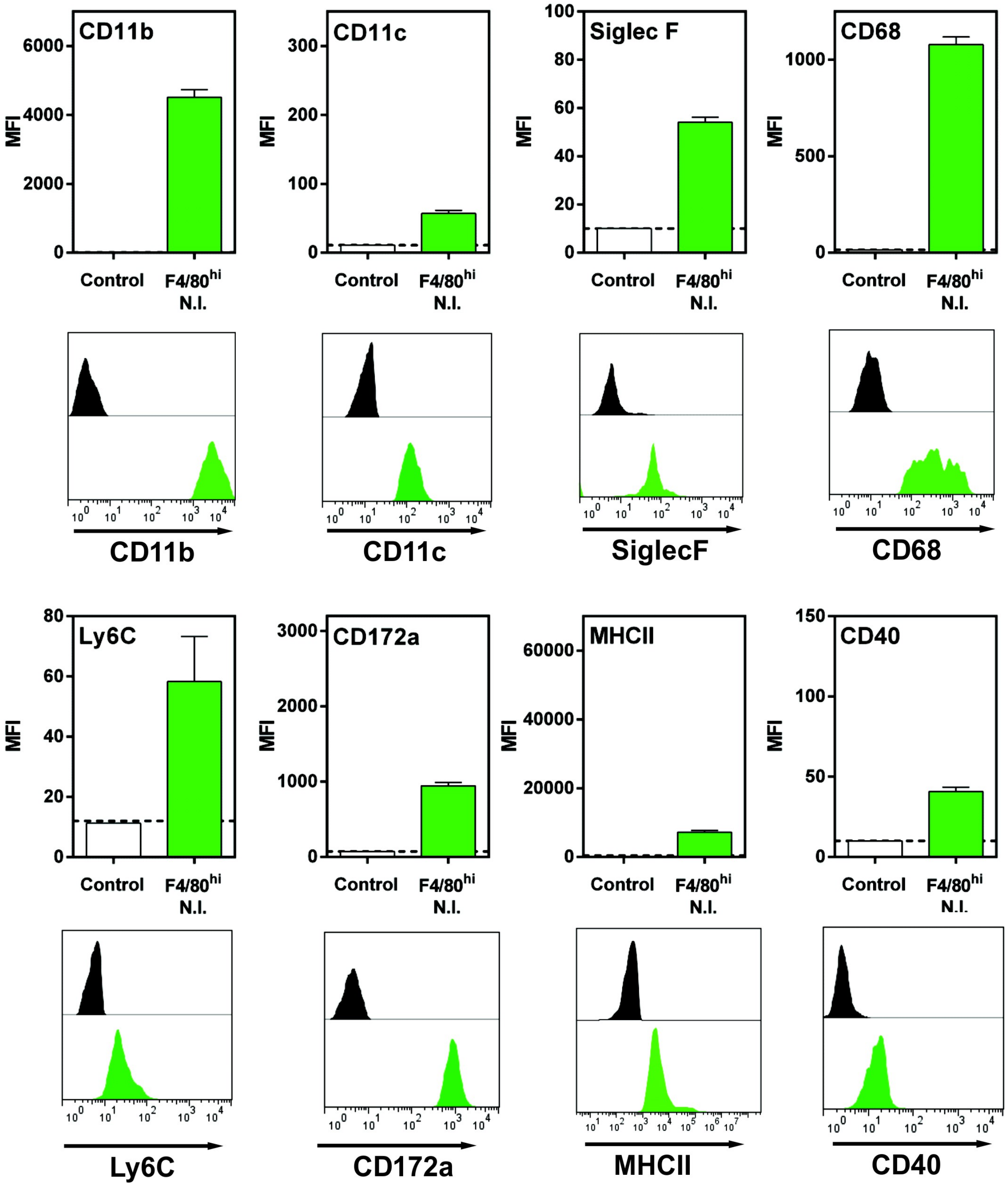

Supplementary Figure 4. Immunephenotyping of HO-1$^-$ F4/80^{hi} in the peritoneum of control animals. A) HO-1$^-$ F4/80^{hi} cells (green) from PECs of non-infected mice were evaluated for the expression of different cell markers by flow cytometry. Cells from the peritoneal cavity from naive mice were stained with CD11b- CD11c-, MHCII, CD40, SIRPa, CD68-, Ly6C- or Siglec-F- specific antibodies and evaluated by flow cytometry. Results are representative of three independent experiments is shown. Control corresponds to unstained cells.
